# Supplementary material for: Current-induced switching of a van der Waals ferromagnet at room temperature
Source: Nat Commun. 2024 Feb 19;15:1485. doi: 10.1038/s41467-024-45586-4 (PMC10876566; doi:10.1038/s41467-024-45586-4)
Supplement: Supplementary file 1 — Supplementary Information [file 41467_2024_45586_MOESM1_ESM.pdf]

Supplementary Information for

Current-induced switching of a van der Waals ferromagnet at room temperature

Shivam N. Kajale[1], Thanh Nguyen[2], Corson A. Chao[3], David C. Bono[3], Artittaya Boonkird[2], Mingda Li[2], Deblina Sarkar[1*]

[1] MIT Media Lab, Massachusetts Institute of Technology, Cambridge, MA, 02139, USA
[2] Department of Nuclear Science and Engineering, Massachusetts Institute of Technology, Cambridge, MA, 02139, USA
[3] Department of Materials Science and Engineering, Massachusetts Institute of Technology, Cambridge, MA, 02139, USA
These authors contributed equally: Shivam N. Kajale, Thanh Nguyen
* deblina@mit.edu

Section 1: Current distribution across Pt and FGaT

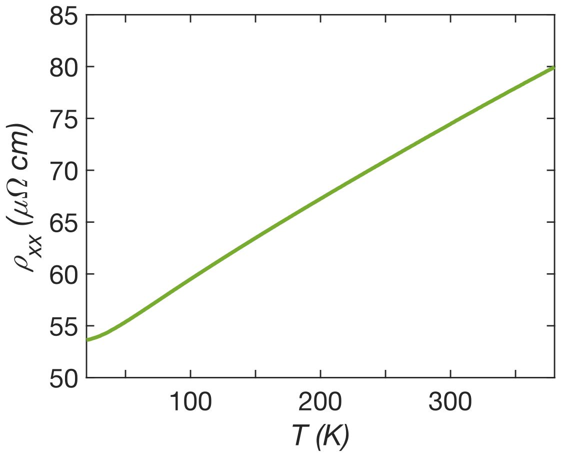

Figure 1: Longitudinal resistivity of 6 nm Pt on Si/SiO_2.

For a FGaT/Pt bilayer Hall bar, of width w and length l, upon equating voltage drop across both materials (parallel current channels), we have,

$$I_{Pt}R_{Pt} = I_{FGaT}R_{FGaT}$$

$$\therefore \frac{I_{Pt}}{I_{FGaT}} = \frac{R_{FGaT}}{R_{Pt}} = \frac{\frac{\rho_{FGaT}l}{wt_{FGaT}}}{\frac{\rho_{Pt}l}{wt_{Pt}}} = \frac{\rho_{FGaT}t_{Pt}}{\rho_{Pt}t_{FGaT}}$$

where, $\rho_{Pt}, \rho_{FGaT}, t_{Pt}$ and t_{FGaT} are the resistivities of Pt, FGaT and the thicknesses of Pt and FGaT in the device, respectively.

$$\therefore \frac{I_{Pt}}{I_{total}} = \frac{\rho_{FGaT}t_{Pt}}{\rho_{Pt}t_{FGaT} + \rho_{FGaT}t_{Pt}}$$

Section 2: Current induced switching - Additional FGaT/Pt devices

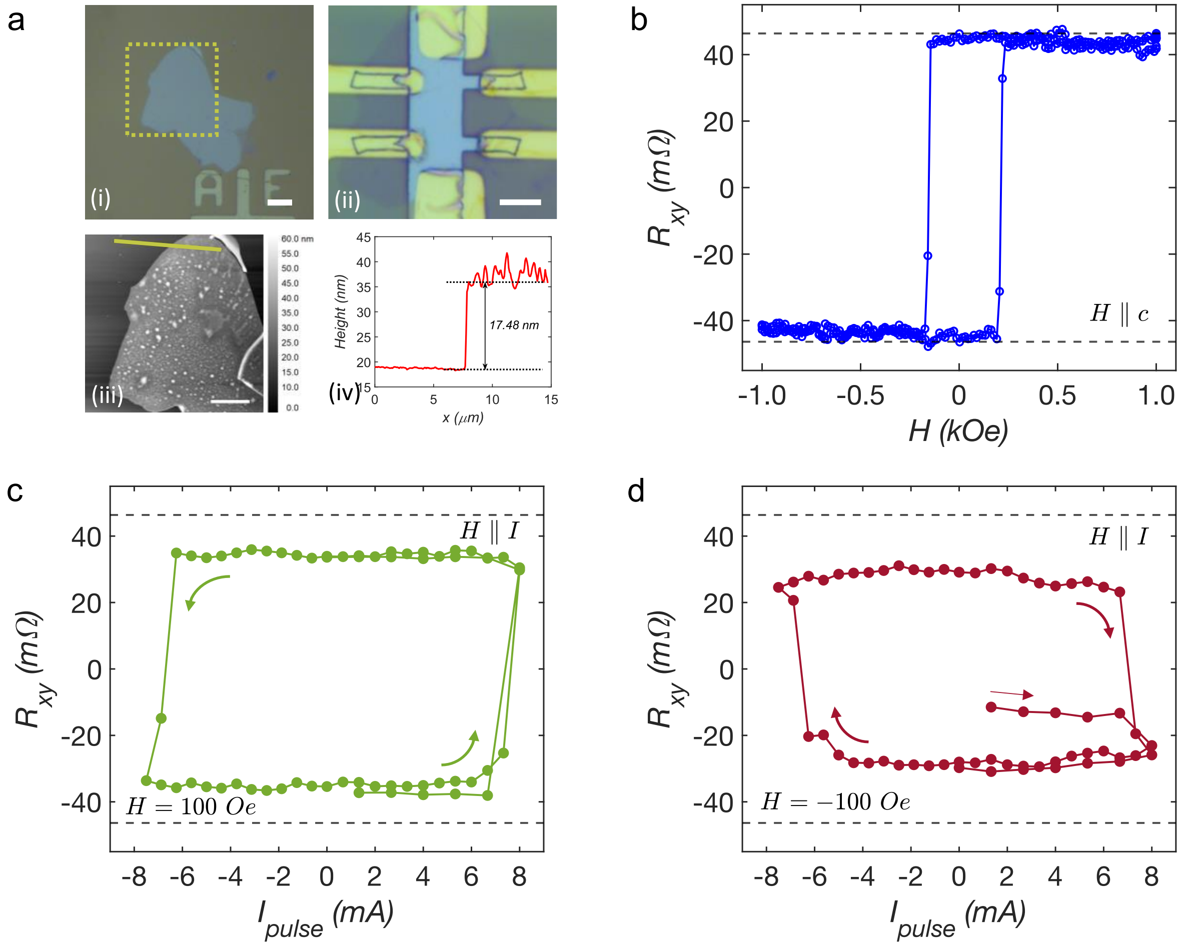

Figure 2: (a) Morphological data for device D2 – Optical image of (i) the FGaT flake before device fabrication and (ii) the corresponding fabricated device. (iii) AFM topographical image of the FGaT flake corresponding to the dotted yellow square in (i), and (iv) AFM height profile along the solid yellow line in (iii). Scale bars – 5 μm **(b)** Anomalous Hall effect hysteresis plot for the device with field swept out-of-plane (OOP) ($H \parallel c$) **(c, d)** Current-driven magnetization switching loops for the device, in the presence of an externally applied in-plane magnetic field parallel to the current injection ($H \parallel I$) and magnitude (c) +100 Oe and (d) -100 Oe. Black dashed line in (b, c, d) are visual guides for the maximum anomalous Hall resistance observable in the device at 300 K. All data corresponds to 300 K.

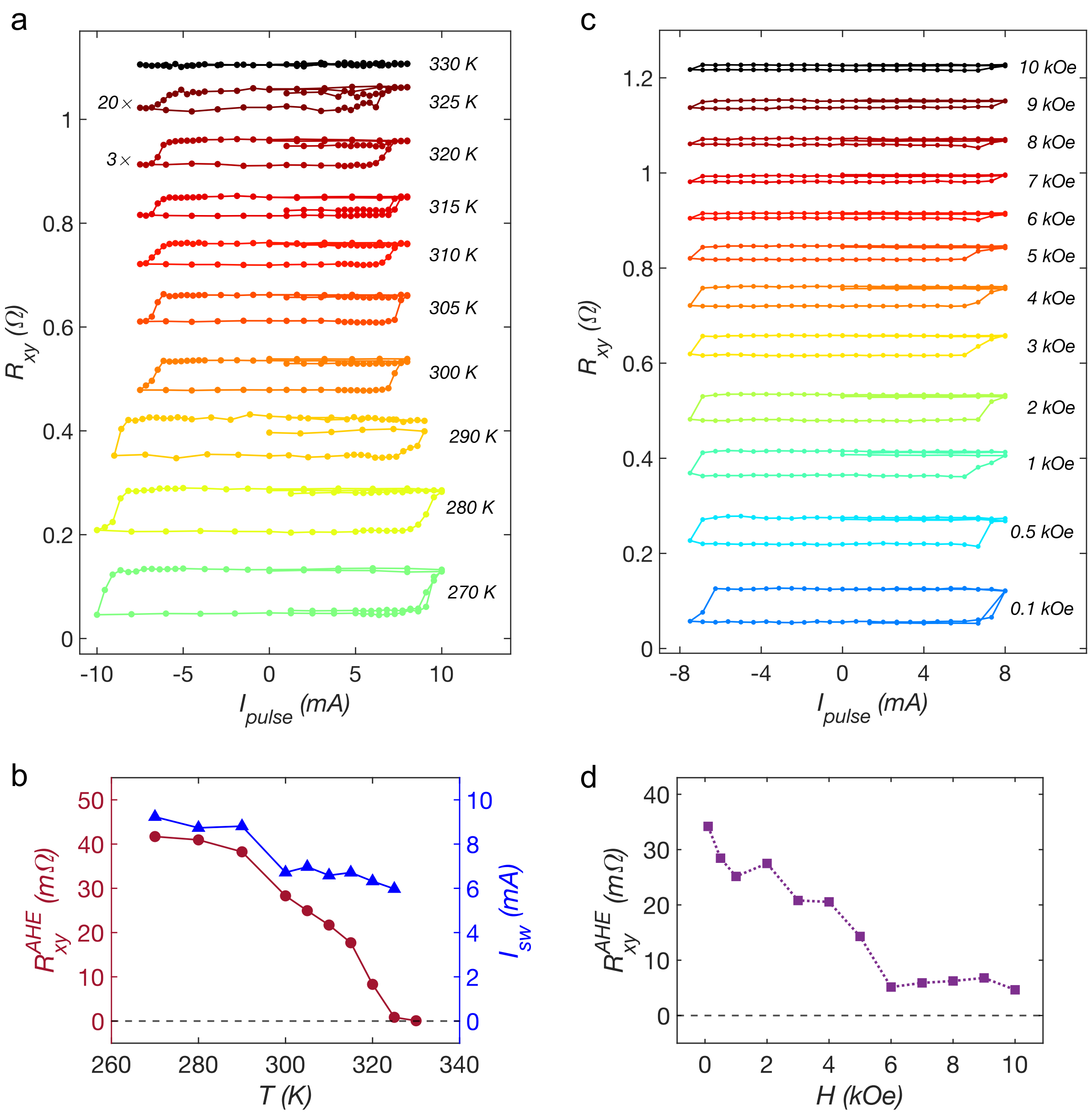

Figure 3: (a) Temperature dependent current-induced switching loops for the device D2, under 500 Oe external field applied parallel to the current injection direction. Data offset along y-axis, and scaled for 320 K and 325 K, for clarity. **(b)** Temperature dependence of switched anomalous Hall resistance (R_{xy}^{AHE}) and threshold switching current (I_{sw}). R_{xy}^{AHE} decreases with increasing temperature as can be expected due to decreasing magnetization, until no clear switching is observed at 330 K, which coincides with the Curie temperature of our FGaT nanosheets. Switching current for a particular temperature is estimated by fitting sigmoid functions to the positive and negative transition edge of the loop and taking the average of the two transition points. A gradual decrease in switching current is observed with increasing temperature. **(c)** Current-induced switching loops for the device under increasing external magnetic field applied parallel to the current, at 300 K. **(d)** Variation of the switched anomalous Hall resistance (R_{xy}^{AHE}) with externally applied field ($H \parallel I$), at 300 K.

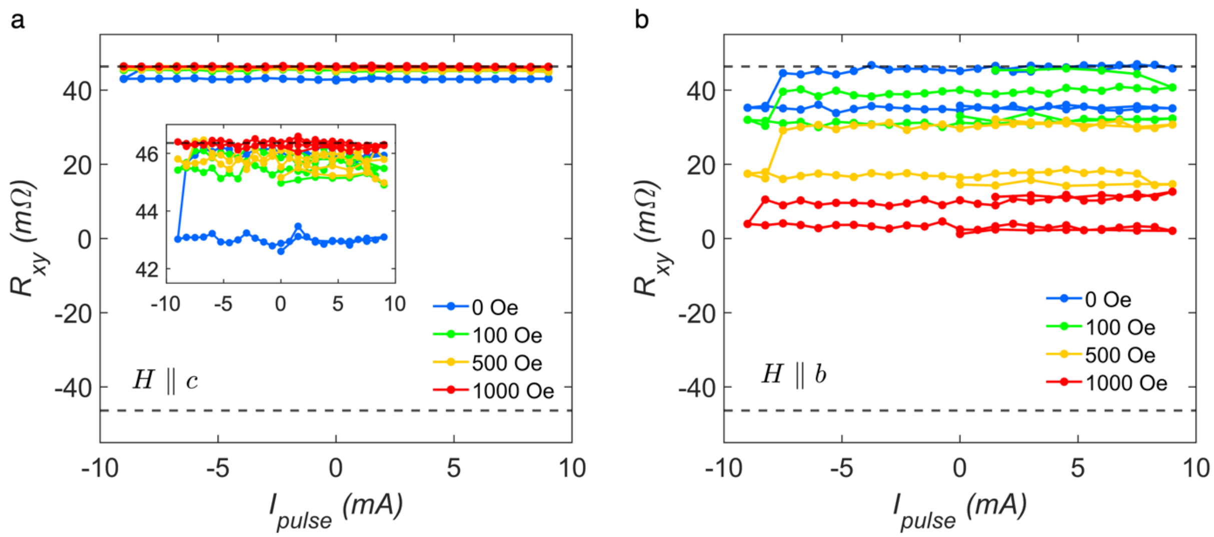

Figure 4: (a) Response of the device D2 to current pulsing when the external field is applied out-of-plane ($H \parallel c$). Inset: Enlarged view of the data at high resistances. Under 0 Oe field, we observe a small drop in R_{xy} at maximum current corresponding to a slight demagnetization. For field ≥100 Oe, no such demagnetization is observed, as the OOP field keeps the magnetization aligned OOP. **(b)** Response of D2 to current pulsing when the external field is applied in-plane and orthogonal to the current injection direction, i.e. $H \parallel b$. In this case, the field neither assists the deterministic SOT switching, nor does it help saturate the magnetization OOP. Thus, the device which is initially saturated along +z direction, gradually demagnetizes under subsequent pulsing cycles. No clear effect of field variation could be observed. Measurements were done sequentially under 0, 100, 500, and 1000 Oe respectively.

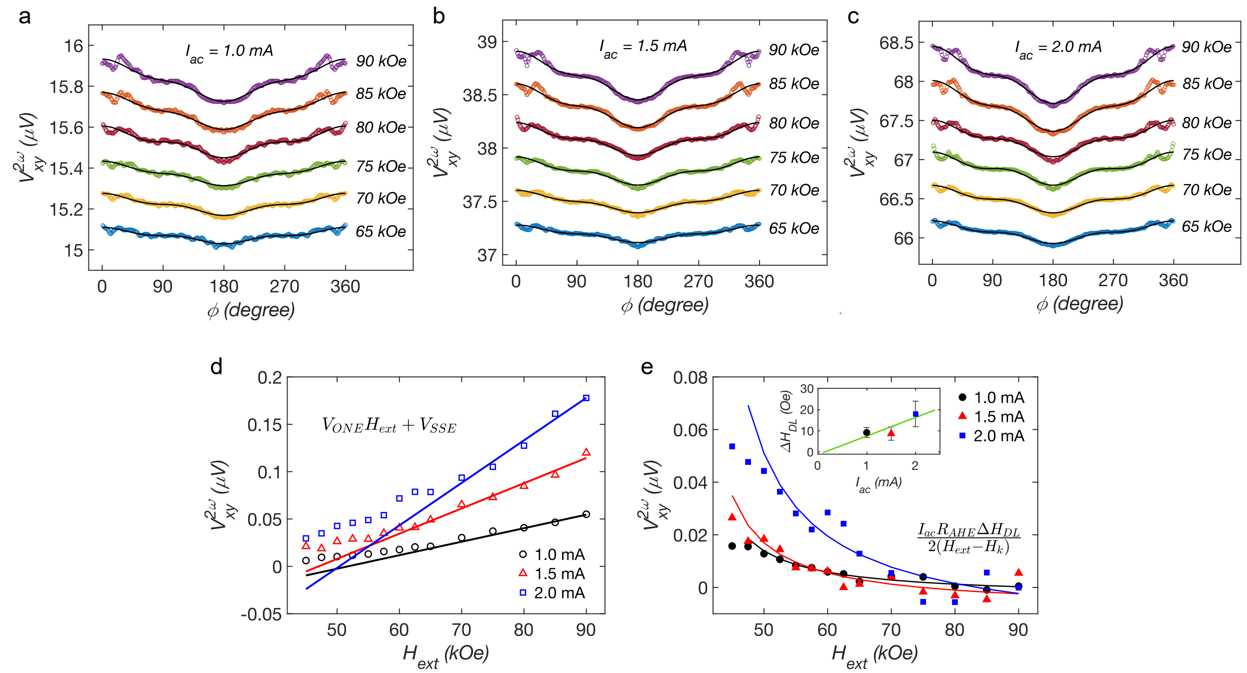

Figure 5: Spin-orbit torque efficiency of D2. (a, b, c) The second harmonic Hall voltage, $V_{\mathrm{xy}}^{2\omega}$, measured under in-plane magnetic field rotation for (a) $I_{\mathrm{ac}} = 1.0$ mA, (b) $I_{\mathrm{ac}} = 1.5$ mA, and (c) $I_{\mathrm{ac}} = 2.0$ mA. Solid black lines fit to equation (2). Data offset in y-axis for clarity. **(d)** Hollow symbols represent the amplitude of $\cos\phi$ components of $V_{\mathrm{xy}}^{2\omega}$ in equation (3). Solid lines are fits for the linear, thermal contribution to $V_{\mathrm{xy}}^{2\omega}$ from ordinary Nernst effect and spin Seebeck effect. **(e)** Anti-damping-like field contribution to $V_{\mathrm{xy}}^{2\omega}$ (solid symbols) and their theoretical fits (solid lines), with $H_{\mathrm{k}} = 38$ kOe. Inset: ΔH_{DL} extracted for the three current amplitudes, and their fitting line, with a near zero y-intercept. Error bars represent a 95% confidence interval. The damping-like field is estimated to be 4.66×10^{-10} Oe $A^{-1}m^2$, and the anti-damping-like spin-orbit torque efficiency is ξ_{DL} = 0.098. Measurements are performed at 300 K. Refer to main text for fitting equations.

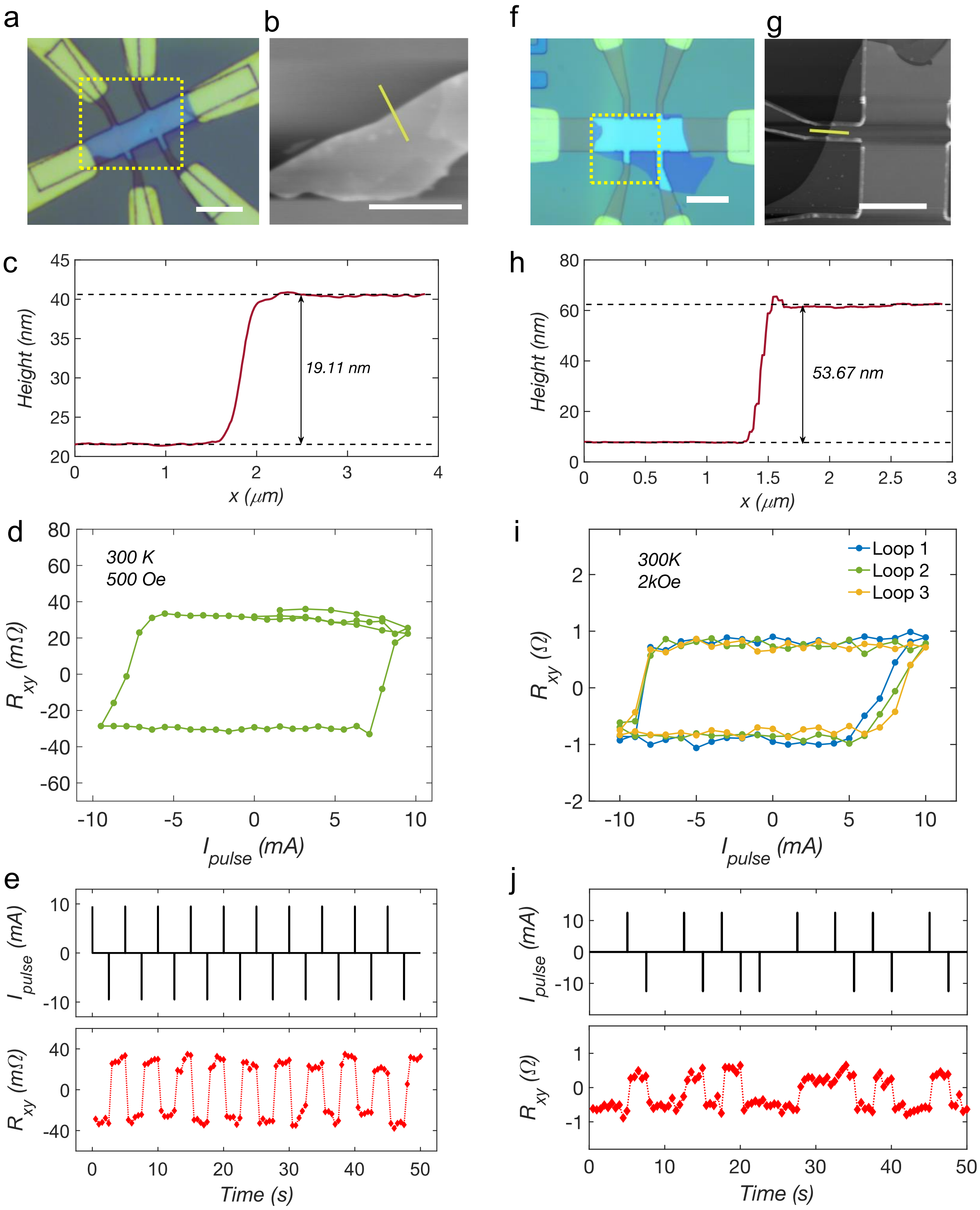

Figure 6: (a) Optical image of device D3. **(b)** AFM topography image of the corresponding FGaT flake in the dotted yellow box in (a) before device fabrication, and **(c)** AFM height profile along the solid yellow line in (b). **(d)** Current-induced switching loop in D3 recorded through anomalous Hall resistance measurements, **(e)** and robust magnetization switching observed in response to a train of current pulses, 1 ms long and 9.5 mA in magnitude. (d, e) recorded at 300 K with 500 Oe external field applied parallel to current. **(f)** Optical image of device D4. **(g)** AFM

topography image of the corresponding FGaT flake in the dotted yellow box in (f), and **(h)** AFM height profile along the solid yellow line in (g). **(i)** Current-induced switching loop in D3 recorded through anomalous Hall resistance measurements, **(j)** and robust magnetization switching observed in response to a train of current pulses, 1 ms long and 12 mA in magnitude. (i, j) recorded at 300 K with 2 kOe external field applied parallel to current.

Table 1: Summary of FGaT/Pt devices reported in this study.

**Device**	**FGaT thickness (nm)**	I_{sw} **(mA)**	J_{sw} **(A m^{-2})**	$\Delta H_{DL}/J_c$ **(Oe/Am^{-2})**	ξ_{DL}
D1	57.9	5.4	1.69×10^{10}	1.34×10^{10}	0.093
D2	17.5	6.7	4.23×10^{10}	4.66×10^{10}	0.098
D3	19.1	8.5	9.54×10^{10}	-	-
D4	53.7	9.0	2.15×10^{10}	-	-

Section 3: Current induced switching in vdW magnetic materials

Ferro-magnet	Spin-Hall material	T (K)	J_{sw} (Am^{-2})	$\Delta H_{DL}/J_c$ (Oe/Am^{-2})	ξ_{DL}	Reference
Fe_3GeTe_2	Pt	180	2.5×10^{11}		0.14	Alghamdi et al.[1]
Fe_3GeTe_2	Pt	100	9.25×10^{10}	5.34×10^{-9}	0.12	Wang et al.[2]
Fe_3GeTe_2	$(Bi,Sb)_2Te_3$	180	1.7×10^{10}			Fujimura et al.[3]
Fe_3GeTe_2	WTe_2	200	6.6×10^{10}			Kao et al.[4]
Fe_3GeTe_2	WTe_2	150	3.9×10^{10}		4.6	Shin et al.[5]
Fe_3GeTe_2	WTe_2	120	5.9×10^{10}			Wang et al.[6]
$CrTe_2$	$ZrTe_2$	50	1.8×10^{11}			Ou et al.[7]
$Cr_2Ge_2Te_6$	Pt		1.5×10^{9}* (insulator)	2×10^{-10}	0.25	Gupta et al.[8]
Fe_3GaTe_2	Pt	300	1.69×10^{10}	1.34×10^{-10}	0.093	This work.

*The FM is an insulator and hence not suitable for developing devices like magnetic tunnel junctions

References

S1. Alghamdi, M., Lohmann, M., Li, J., Jothi, P. R., Shao, Q., Aldosary, M., Su, T., Fokwa, B. P. T. & Shi, J. Highly Efficient Spin-Orbit Torque and Switching of Layered Ferromagnet Fe3GeTe2. *Nano Lett.* **19,** 4400–4405 (2019).

S2. Wang, X., Tang, J., Xia, X., He, C., Zhang, J., Liu, Y., Wan, C., Fang, C., Guo, C., Yang, W., Guang, Y., Zhang, X., Xu, H., Wei, J., Liao, M., Lu, X., Feng, J., Li, X., Peng, Y., Wei, H., Yang, R., Shi, D., Zhang, X., Han, Z., Zhang, Z., Zhang, G., Yu, G. & Han, X. Current-driven magnetization switching in a van der Waals ferromagnet Fe3GeTe2. *Sci. Adv.* **5,** aaw8904 (2019).

S3. Fujimura, R., Yoshimi, R., Mogi, M., Tsukazaki, A., Kawamura, M., Takahashi, K. S., Kawasaki, M. & Tokura, Y. Current-induced magnetization switching at charge-transferred interface between topological insulator (Bi,Sb)2Te3 and van der Waals ferromagnet Fe3GeTe2. *Appl. Phys. Lett.* **119,** 032402 (2021).

S4. Kao, I. H., Muzzio, R., Zhang, H., Zhu, M., Gobbo, J., Yuan, S., Weber, D., Rao, R., Li, J., Edgar, J. H., Goldberger, J. E., Yan, J., Mandrus, D. G., Hwang, J., Cheng, R., Katoch, J. & Singh, S. Deterministic switching of a perpendicularly polarized magnet using unconventional spin–orbit torques in WTe2. *Nat. Mater.* **21,** 1029–1034 (2022).

S5. Shin, I., Cho, W. J., An, E. S., Park, S., Jeong, H. W., Jang, S., Baek, W. J., Park, S. Y., Yang, D. H., Seo, J. H., Kim, G. Y., Ali, M. N., Choi, S. Y., Lee, H. W., Kim, J. S., Kim, S. D. & Lee, G. H. Spin–Orbit Torque Switching in an All-Van der Waals Heterostructure. *Adv. Mater.* **34,** 1–7 (2022).

S6. Wang, L., Xiong, J., Cheng, B., Dai, Y., Wang, F., Pan, C., Cao, T., Liu, X., Wang, P., Chen, M., Yan, S., Liu, Z., Xiao, J., Xu, X., Wang, Z., Shi, Y., Cheong, S. W., Zhang, H., Liang, S. J. & Miao, F. Cascadable in-memory computing based on symmetric writing and readout. *Sci. Adv.* **8,** abq6833 (2022).

S7. Ou, Y., Yanez, W., Xiao, R., Stanley, M., Ghosh, S., Zheng, B., Jiang, W., Huang, Y. S., Pillsbury, T., Richardella, A., Liu, C., Low, T., Crespi, V. H., Mkhoyan, K. A. & Samarth, N. ZrTe2/CrTe2: an epitaxial van der Waals platform for spintronics. *Nat. Commun.* **13,** 2972 (2022).

S8. Gupta, V., Cham, T. M., Stiehl, G. M., Bose, A., Mittelstaedt, J. A., Kang, K., Jiang, S., Mak, K. F., Shan, J., Buhrman, R. A. & Ralph, D. C. Manipulation of the van der Waals Magnet Cr2Ge2Te6by Spin-Orbit Torques. *Nano Lett.* **20,** 7482–7488 (2020).
